# Expression and clinical significance of the phosphatidylinositol 3-kinase/protein kinase B signal transduction pathway in non-small cell lung carcinoma

**DOI:** 10.3892/ol.2014.2167

**Published:** 2014-05-22

**Authors:** AI-GUI JIANG, HONG YU, JIAN-AN HUANG

**Affiliations:** 1 Department of Respiratory Medicine, Taizhou People’s Hospital, Taizhou, Jiangsu 225300, P.R. China; 2 Department of Pathology, Taizhou People’s Hospital, Taizhou, Jiangsu 225300, P.R. China; 3 Department of Respiratory Medicine, The First Affiliated Hospital of Soochow University, Suzhou, Jiangsu 215006, P.R. China

**Keywords:** non-small cell lung carcinoma, prognosis, phosphatidylinositol 3-kinase, protein kinase B

## Abstract

The overactivation of the phosphatidylinositol 3-kinase (PI3K)/protein kinase B (Akt) signal transduction pathway has been examined in various carcinomas and is reported to be significantly correlated with prognosis. However, little is known with regard to the PI3K/Akt signal transduction pathway in advanced non-small cell lung carcinoma (NSCLC). The present study investigated the expression of PI3K and phosphorylated (p)-Akt protein and its clinical significance in NSCLC. The clinical records of 157 patients with NSCLC (70 stage I–IIIA and 87 stage IIIB–IV cases), consisting of 75 cases of squamous cell carcinoma and 82 cases of adenocarcinoma, together with 30 resected lung cancer tumor-adjacent tissue samples, were retrospectively evaluated. PI3K and p-Akt expression in the NSCLC and tumor-adjacent tissues were measured using an immunohistochemical method, and its correlation with the clinicopathological data and prognosis in advanced NSCLC was evaluated. PI3K and p-Akt expression was significantly higher in the cancer tissues (χ^2^=14.8455; P=0.001) than in the tumor-adjacent tissues (χ^2^=14.2615; P=0.001). The overexpression of p-Akt in stage I–IIIA NSCLC was associated with lymph node metastasis (χ^2^=6.1189; P=0.013) and tumor-node-metastasis (TNM) stage (χ^2^=8.9752; P=0.011), however, no correlation was observed with gender, age, pathological type and histological grade. The overexpression of p-Akt in stage IIIB–IV NSCLC was only associated with TNM stage (χ^2^=5.7501; P=0.016), and no correlation was observed with gender, age, pathological type, histological grade and Eastern Cooperative Oncology Group (ECOG) performance status (PS). The overexpression of PI3K was not found to correlate with the aforementioned clinicopathological variables in all patients. Survival was significantly improved in advanced NSCLC with PI3K- and p-Akt-negative expression compared with PI3K- and p-Akt-positive expression [P13K: 17.70 months (95% confidence interval (CI), 15.11–20.28 months) vs. 13.43 months (95% CI, 11.83–15.02 months); P=0.004; and p-Akt: 17.13 months (95% CI, 14.93–19.34 months) vs. 13.07 months (95% CI, 11.32–14.82 months); P=0.007]. Multivariate analysis showed that PI3K [hazard ratio (HR)=2.143; 95% CI, 1.211–3.790; P=0.009], p-Akt (HR=1.991; 95% CI, 1.009–3.927; P=0.047), TNM stage (HR=4.788; 95% CI, 2.591–8.848; P=0.001) and ECOG-PS (HR=3.272; 95% CI, 1.701–6.296; P=0.001) were independent predictors for survival in stage IIIB–IV NSCLC. These results indicated that p-Akt overexpression closely correlates with factors of an unfavorable prognosis in NSCLC. PI3K and p-Akt overexpression are independent markers of a poor prognosis in advanced NSCLC.

## Introduction

Non-small cell lung carcinoma (NSCLC) is one of the leading causes of cancer-related mortality worldwide, despite considerable progress in surgery, chemotherapy, radiotherapy and biological targeted therapy (1). Recent research in the last decade has shown that these therapies predominantly improve a patient’s quality of life; the overall five-year survival rate for patients with such tumors is <15% (2). As a consequence, research into novel prognostic biomarkers and therapeutic target structures in NSCLC remains a focus of attention.

Phosphatidylinositol 3-kinase (PI3K)/protein kinase B (Akt) (of the PI3K/Akt signaling pathway) have been shown to be involved in the regulation of cell proliferation and apoptosis, and are key to the initiation and progression of malignancies, enhancing cell survival by the stimulation of cell proliferation and the inhibition of apoptosis (3,4). More recent studies have identified the activation of the PI3K/Akt signaling pathway in several types of human cancer, including brain glioma (5), breast cancer (6) and pancreatic cancer (7). In addition, the high expression of PI3K and phosphorylated (p)-Akt are often associated with a poor prognosis. PI3K and p-Akt expression have also been detected in the early stage of NSCLC (8–13), however, their clinical significance in operative NSCLC remains controversial (8,9,13).

One study has reported that the antigen expression of PI3K and p-Akt may be associated with the tumor-node-metastasis (TNM) stage of NSCLC (8). However, to the best of our knowledge, there have been no studies on PI3K and p-Akt expression in advanced NSCLC and the association with prognosis. In the present retrospective study, the correlations between the antigen expression of PI3K and p-Akt, and the clinicopathological data of NSCLC patients and the prognosis in advanced NSCLC were evaluated.

## Patients and methods

### Patients

The clinical records of 157 patients (110 males and 47 females; mean age, 57.3 years; range, 46–72 years) with NSCLC (70 stage I–IIIA and 87 stage IIIB–IV cases), who were admitted to the Taizhou People’s Hospital (Taizhou, Jiangsu, China) between June 2008 and June 2010 were retrospectively evaluated. In total, 75 cases of squamous cell carcinoma (SCC), 82 cases of adenocarcinoma (AdC) and 30 resected lung cancer tumor-adjacent tissue samples were obtained. Stage I–IIIA patients were confirmed based on the histopathology report following surgery, while stage IIIB–IV patients were confirmed using computed tomography-guided percutaneous or bronchoscopic lung biopsies. The patients were divided into stages I, II, IIIA, IIIB and IV tumor groups, according to the TNM system (14). Patients with advanced NSCLC were excluded from the study if they had received prior chemotherapy or radiotherapy, or if they had no definitive histological diagnosis, a poor performance status (PS) [Eastern Cooperative Oncology Group (ECOG)-PS of ≥3), brain tumor metastasis or a disease other than lung cancer that may have affected survival, including cardiac dysfunction, renal insufficiency, liver cirrhosis or concomitant malignancy. This study was approved by the Ethics Committee of Taizhou People’s Hospital and was performed according to the Declaration of Helsinki. Written informed consent was obtained from the family of each patient.

### Immunohistochemistry

Paraffin-embedded tissue blocks were cut into 4-μm sections, and representative sections were analyzed immunohistochemically (EliVision™ Plus IHC kit; Wuhan Boster Biological Engineering Co., Ltd. Wuhan, China) for PI3K and p-Akt (1:200; mouse polyclonal antibody; Miltenyi Biotec, San Diego, CA, USA). Briefly, the sections were dewaxed in xylene and rehydrated in ethanol through graded concentrations of alcohol. Endogenous peroxidase activity was blocked by incubating the sections in 5% hydrogen peroxide in absolute methanol at room temperature for 10 min. Antigen retrieval was performed in a microwave oven for two cycles of 10 min each. Primary antibodies were applied for 1 h at room temperature and the sections were washed three times with 0.05 M Tris-buffered saline [TBS (pH 7.2)]. Next, 50 μl immunoglobulin G/horseradish peroxidase secondary antibody (Wuhan Boster Biological Engineering Co., Ltd.) was added, followed by incubation for 30 min at room temperature. The sections were washed three times with TBS and the reaction products were visualized with diaminobenzidine (DAB kit; Wuhan Boster Biological Engineering Co., Ltd.). The sections were counterstained with hematoxylin and eosin (Wuhan Boster Biological Engineering Co., Ltd.), dehydrated and evaluated under a light microscope (DM3000, Leica, Mannheim, Germany).

### Immunohistochemistry scoring

Positive staining for PI3K and p-Akt was assessed in 10 high-power fields of each tumor by two independent pathologists using light microscopy in a blinded manner. The mean rate of positive tumor cells was calculated. PI3K and p-Akt expression was evaluated for each tissue sample by calculating a total immunostaining score as the product of the proportion and intensity scores. The proportion score described the estimated fraction of positively-stained tumor cells (0, none; 1, ≤10%; 2, 10–50%; 3, 51–80%; and 4, ≥80%), while the intensity score represented the estimated staining intensity (0, no staining; 1, weak; 2, moderate; and 3, strong). Thus, the total score ranged between 0 and 12. The positive and negative expression values of PI3K and p-Akt were defined as scores of >4 and ≤4, respectively.

### Follow-up

The stage IIIB–IV NSCLC patients were followed up from the date of pathological diagnosis until mortality or the last follow-up to the outpatient department. At the time of the last follow-up, 80 patients (92%) had succumbed to the disease, and seven patients (8%) were lost to follow-up or had succumbed to other causes.

### Statistical analysis

The statistical analysis was performed using the SPSS 13.0 software (SPSS, Inc., Chicago, IL, USA). Correlations between PI3K and p-Akt immunostaining and the clinicopathological parameters, including gender, age, pathological type, histologic grade, lymph node metastasis, TNM stage and ECOG-PS, were analyzed using χ^2^ and Fisher’s exact tests. The overall survival (OS) of the stage IIIB–IV NSCLC patients was calculated from the date of diagnosis to the date of the last follow-up or mortality. The cases lost to follow-up or mortality due to any other causes were defined as censored data for the analysis of the survival rates. The survival curves were plotted using the Kaplan-Meier method, and P-values were calculated using the log-rank test. A multivariate analysis was performed using Cox-proportional hazards model to identify independent prognostic factors. P≤0.05 was considered to indicate a statistically significant difference.

## Results

### PI3K and p-Akt expression in stage I–IIIA NSCLC and tumor-adjacent tissues

**Figure 1:**
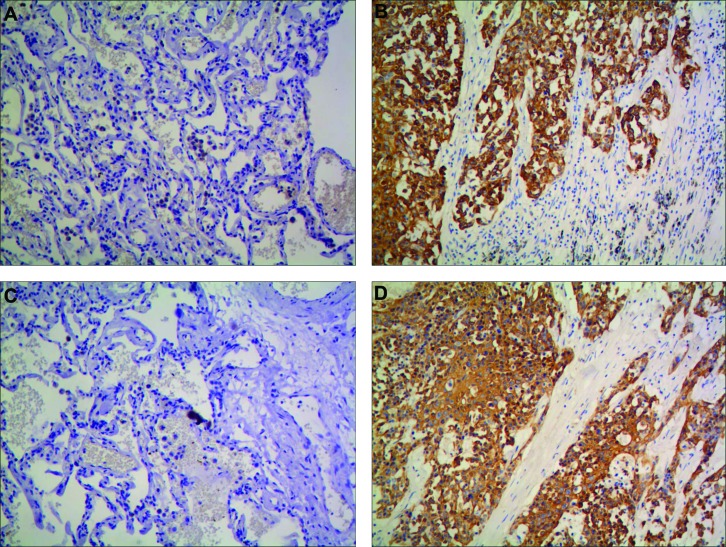
PI3K and p-Akt expression in stage I–IIIA (A and C) tumor-adjacent tissues and (B and D) squamous cell carcinoma. (A) PI3K(−); (B) PI3K(+); (C) p-Akt(−); and (D) p-Akt(+). (Stain, hematoxylin and eosin; magnification, ×200). PI3K, phosphatidylinositol 3-kinase; p-Akt, phosphorylated protein kinase B.

Staining for PI3K and p-Akt demonstrated diffuse brown particles of varying thickness patterns localized predominantly in the membrane and cytoplasm of the cancer cells, however, staining was also occasionally identified in the nucleus. PI3K and p-Akt expression was absent or infrequent in the tumor-adjacent tissues. Furthermore, PI3K and p-Akt overexpression was detected in 58.6 (41/70) and 50.0% (35/70) of the tumors, which was higher than in the tumor-adjacent tissues for PI3K [16.7% (5/30); χ^2^=14.8455; P<0.001] and p-Akt [10.0% (3/30); χ^2^=14.2615; P=0.001] (Fig. 1).

### PI3K and p-Akt expression in stage IIIB–IV NSCLC tissues

**Figure 2:**
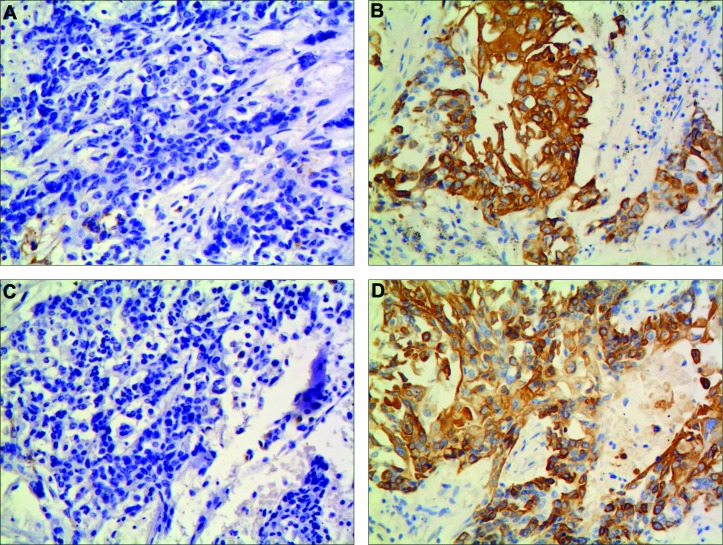
PI3K and p-Akt expression in stage IIIB–IV squamous cell carcinoma. (A) PI3K(−); (B) PI3K(+); (C) p-Akt(−); and (D) p-Akt(+). (Stain, hematoxylin and eosin; magnification, ×400). PI3K, phosphatidylinositol 3-kinase; p-Akt, phosphorylated protein kinase B.

**Figure 3:**
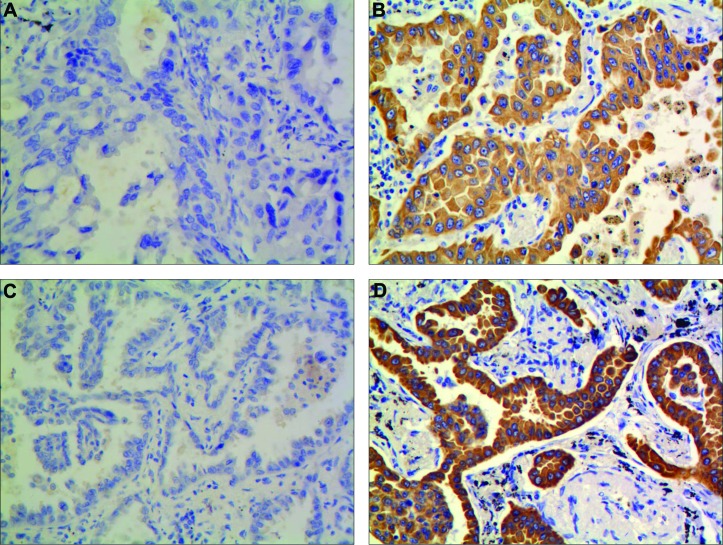
PI3K and p-Akt expression in stage IIIB–IV adenocarcinoma. (A) PI3K(−); (B) PI3K(+); (C) p-Akt(−); and (D) p-Akt(+). (Stain, hematoxylin and eosin; magnification, ×400). PI3K, phosphatidylinositol 3-kinase; p-Akt, phosphorylated protein kinase B.

No significant difference was observed between the staining location of PI3K and p-Akt in the stage IIIB–IV and I–IIIA NSCLC tissues. In the stage IIIB–IV tissues, the positive expression rate of PI3K and p-Akt was 58.6 (51/87) and 45.9% (40/87), respectively, which did not differ significantly with regard to the expression in the stage I–IIIA NSCLC tissues (Figs. 2 and 3).

### Correlation between PI3K and p-Akt expression and the clinicopathological variables

No significant difference was identified in p-Akt overexpression in stage I–IIIA NSCLC patients with regard to patient gender, age, pathological type or degree of differentiation. However, p-Akt overexpression in stage I–IIIA NSCLC was found to significantly correlate with lymph node metastasis (χ^2^=6.1189; P=0.013) and TNM stage (χ^2^=8.9752; P=0.011). Furthermore, no significant difference was identified in p-Akt overexpression in stage IIIB–IV NSCLC with regard to patient gender, age, pathological type, degree of differentiation or ECOG-PS. However, p-Akt overexpression in stage IIIB–IV NSCLC was found to significantly correlate with TNM stage (χ^2^=5.7501; P=0.016). The overexpression of PI3K was not correlated with the aforementioned clinicopathological variables in all patients (Tables I and II).

### Correlation between PI3K and p-Akt expression and prognosis in stage IIIB–IV NSCLC

**Figure 4:**
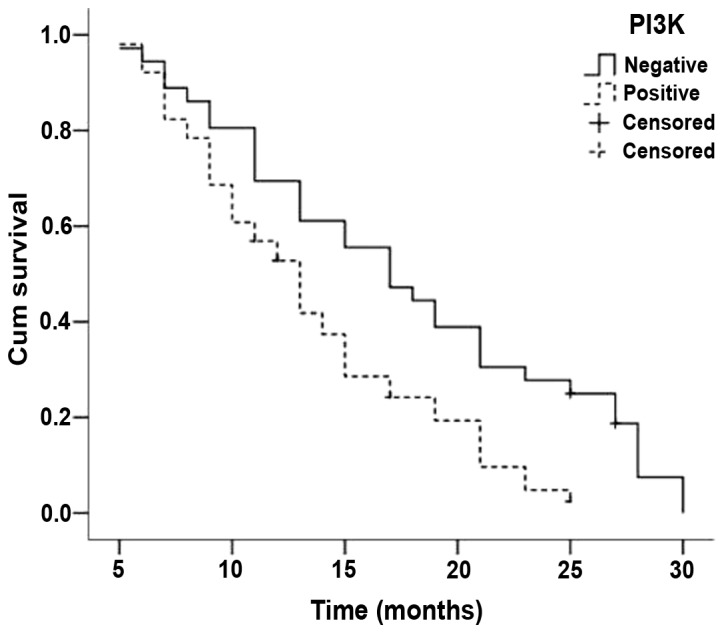
Kaplan-Meier curves for stage IIIB–IV non-small cell lung carcinoma (NSCLC) patients with PI3K-positive and -negative expression (log-rank, χ^2^=8.347 and P=0.004). Cum, cumulative; PI3K, phosphatidylinositol 3-kinase.

**Figure 5:**
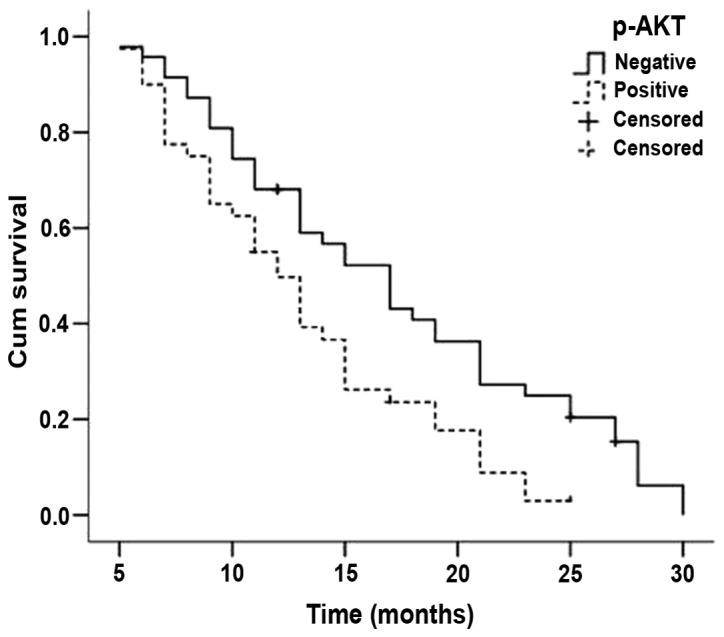
Kaplan-Meier curves for stage IIIB–IV non-small cell lung carcinoma (NSCLC) patients with p-Akt-positive and -negative expression (log-rank, χ^2^=7.192 and P=0.007). Cum, cumulative; Akt, protein kinase B.

The median OS time of all the patients was 15.33 months (95% CI, 13.81–16.85), and survival time was significantly improved in the advanced NSCLC patients with PI3K- and p-Akt-negative expression compared with the patients with positive expression [P13K: 17.70 months (95% CI, 15.11–20.28 months) vs. 13.43 months (95% CI, 11.83–15.02 months); P=0.004; and p-Akt: 17.13 months (95% CI, 14.93–19.34 months) vs. 13.07 months (95% CI, 11.32–14.82 months); P=0.007]. Multivariate analysis showed that PI3K (HR=2.143; 95% CI, 1.211–3.790; P=0.009), p-Akt (HR=1.991; 95% CI, 1.009–3.927; P=0.047), TNM stage (HR=4.788; 95% CI, 2.591–8.848; P=0.001) and ECOG-PS (HR=3.272; 95% CI, 1.701–6.296; P=0.001) were independent predictors for survival in stage IIIB–IV NSCLC patients (Figs. 4 and 5; Table III).

## Discussion

The PI3K/Akt signaling pathway is pivotal in the initiation and progression of malignancies, enhancing cell survival by stimulating cell proliferation, inhibiting apoptosis, promoting tumor angiogenesis and enhancing resistance to chemotherapy and radiotherapy (15,16). Signaling is predominantly activated by growth factor receptor tyrosine kinases (17), which in turn converts membrane-bound phosphatidylinositol 4,5-bisphosphonate to phosphatidylinositol 3,4,5-trisphosphate, which subsequently activates Akt by phosphorylation. p-Akt acts to promote cell proliferation and survival by antagonizing and inactivating various components of the apoptotic cascade, including Bcl-2 (18), caspase-9 (19), glycogen synthase kinase 3 (20) and Forkhead transcription factor family members (21). p-Akt has also been shown to promote cell proliferation by regulating the stability of cyclin D1 (22) or activating mTOR (23). Furthermore, a previous study revealed that p-Akt is extremely important in the regulation of angiogenesis and metastasis in various types of human malignancy (24).

More recent studies have identified the activation of the PI3K/Akt signaling pathway in several types of human cancer, including brain glioma (5), breast cancer (6) and pancreatic cancer (7). In addition, the high expression of PI3K and p-Akt are often associated with a poor prognosis. PI3K and p-Akt expression have also been detected in the early stage of NSCLC, however, their clinical significance in operative NSCLC remains controversial. Tsurutani *et al* (12) reported that p-Akt was positive in the majority NSCLC specimens, but rarely detected in the surrounding normal tissues, indicating that p-Akt activation is a factor for a poor prognosis for all stages of NSCLC. These results indicated that the activation of the PI3K/Akt signaling pathway is important in the transition from precancerous lesion to malignancy. Balsara *et al* (25) also reported that the overexpression of mTOR, a downstream target of the PI3K/Akt signaling pathway, was significantly higher than the expression in normal lung tissue, and its expression was found to closely correlate with the TNM stage. These findings suggested that the activation of the PI3K/Akt pathway is closely correlated with tumor progression. David *et al* (9) investigated the tumors obtained from 61 patients with NSCLC in three tissue microarrays and found that the positive expression rate of p-Akt was 23% (14/61), indicating that p-Akt is an independent adverse prognostic factor for NSCLC. The expression and clinical significance of p-Akt in operative NSCLC was also confirmed by Al-Saad *et al* (8). Notably, the study also found that the high expression of PI3K in tumor stromal cells is an independent factor for a favorable prognosis for NSCLC. Shah *et al* (13) examined 82 surgically resected stage I–IIIA NSCLC samples for p-Akt by immunohistochemistry and found that high p-Akt levels correlate with high tumor grade, whereby p-Akt is an independent factor for a favorable prognosis for stage I–IIIA NSCLC. Al-Saad *et al* (8) considered that these inconsistent results may be the result of tissue specificity, technical differences, immunohistochemical antibodies obtained from different providers, varying scoring methods, study size and the number of statistical variables entered in the multivariate analysis (8).

In the present study, the clinical records of 70 patients with stage I–IIIA NSCLC were retrospectively evaluated, and it was detected that PI3K and p-Akt expression occurred in the membrane of lung cancer cells, as well as the cytoplasm and occasionally the nucleus. PI3K and p-Akt overexpression were detected in 58.6 and 50.0% of the tumors, which was higher than that observed in the tumor-adjacent tissues. These results revealed that the PI3K/Akt signaling pathway is overactivated in NSCLC and may closely correlate with the initiation and progression of the condition, as observed in previous studies (9,12). The present study also detected that p-Akt overexpression in stage I–IIIA NSCLC was significantly correlated with lymph node metastasis and TNM stage, which revealed that the activation of the PI3K/Akt signaling pathway may be involved in the promotion of cell proliferation, invasion and metastasis in NSCLC.

To the best of our knowledge, no studies have been reported with regard to the correlation between PI3K and p-Akt expression and advanced NSCLC in the English-language literature. The present study revealed that PI3K and p-Akt are detected in advanced NSCLC, however, no significant difference was identified between the staining location and overexpression of PI3K and p-Akt in stage IIIB–IV NSCLC tissues compared with that in stage I–IIIA NSCLC tissues. p-Akt overexpression in advanced NSCLC was found to significantly correlate with TNM stage, which revealed that the activation of the PI3K/Akt signaling pathway may be persistently involved in the progression of NSCLC. Furthermore, the multivariate analysis showed that p-Akt, TNM stage and ECOG-PS were independent and adverse prognostic markers in advanced NSCLC, as observed in the previous studies by David *et al* (9) and Al-Saad *et al* (8), but inconsistent with the study by Shah *et al* (13). We hypothesize that the possible reasons for these contradictory results may include the number of samples and the TNM staging of the selected patients. The present study also revealed that PI3K is an independent and unfavorable prognostic marker in advanced NSCLC, contradictory to the results found in the study by Al-Saad *et al* (9), which revealed that the high expression of PI3K in tumor stromal cells is an independent favorable prognostic factor in NSCLC. The results of the study by Al-Saad *et al* indicate that the stromal overexpression of PI3K enhances stromal cell function by preventing tumor cell proliferation in concert with the assumed function of the immune system.

Due to the limited follow-up time in stage I–IIIA NSCLC, the correlation between PI3K and p-Akt expression and prognosis in stage I–IIIA NSCLC was not analyzed in the present study. With an extension to the follow-up time, this study will be continued in the next phase of research.

In conclusion, the PI3K/Akt signaling pathway is overactivated in NSCLC, and is closely correlated with unfavorable prognostic factors. PI3K and p-Akt are also independent and adverse prognostic markers in advanced NSCLC. The results of the present study further indicate that PI3K and p-Akt may be potential therapeutic targets for NSCLC. However, due to the limitations inherent in retrospective analyses, the prognostic value of PI3K and p-Akt overexpression requires further validation in larger prospective studies.

## Figures and Tables

**Table I:** Correlation between PI3K and p-Akt expression and clinicopathological factors in stage I–IIIA NSCLC.

Characteristics	n	PI3K overexpression		Positive rate, %	χ^2^	P-value	p-Akt overexpression		Positive rate, %	χ^2^	P-value
	
		No, n	Yes, n				No, n	Yes, n			
Gender											
Male	49	18	31	63.3	1.4830	0.223	27	22	44.9	1.7007	0.192
Female	21	11	10	47.6			8	13	61.9		
Age, years											
≥60	50	22	28	56.0	0.4769	0.490	23	27	54.0	1.1200	0.290
<60	20	7	13	65.0			12	8	40.0		
Degree of differentiation											
Low-middle	37	14	23	62.2	0.4170	0.518	18	19	51.4	0.0573	0.811
High	33	15	18	54.5			17	16	48.5		
Pathological type											
SCC	38	15	23	60.5	0.1309	0.717	17	21	55.3	0.9211	0.337
AdC	32	14	18	56.3			18	14	43.8		
TNM stage											
I	12	6	6	50.0	0.8814	0.667	9	3	25.0	8.9752	0.011
II	35	15	20	57.1			20	15	42.9		
IIIA	23	8	15	65.2			6	17	73.9		
Lymph node metastasis											
Yes	26	9	17	65.4	0.7913	0.374	8	18	69.2	6.1189	0.013
No	44	20	24	54.6			27	17	38.6		
PS score											
0–1	52	23	29	55.8	0.6544	0.419	24	28	53.9	1.1966	0.274
2	18	6	12	66.7			11	7	38.9		

PI3K, phosphatidylinositol 3-kinase; p-Akt, phosphorylated-protein kinase B; SCC, squamous cell carcinoma; AdC, adenocarcinoma; NSCLC, non-small cell lung carcinoma; TNM, tumor-node-metastasis; PS, performance status.

**Table II:** Correlation between PI3K and p-Akt expression and clinicopathological factors in stage IIIB–IV NSCLC.

Characteristics^a^	n	PI3K overexpression		Positive rate, %	χ^2^	P-value	p-Akt overexpression		Positive rate %	χ^2^	P-value
	
		No, n	Yes, n				No, n	Yes, n			
Gender											
Male	61	28	33	54.1	1.7209	0.190	31	30	49.2	0.8432	0.358
Female	26	8	18	69.2			16	10	38.5		
Age, years											
≥60	52	25	27	51.9	2.3903	0.122	28	24	46.2	0.0016	0.968
<60	35	11	24	68.6			19	16	45.7		
Degree of differentiation											
Low-middle	49	18	31	63.3	0.9977	0.318	29	20	40.8	1.2029	0.273
High	38	18	20	52.6			18	20	52.6		
Pathological type											
SCC	37	18	19	51.4	1.4025	0.236	21	16	43.2	0.1937	0.660
AdC	50	18	32	64.0			26	24	48.0		
TNM stage											
IIIB	49	23	26	53.1	1.4294	0.232	32	17	34.7	5.7501	0.016
IV	38	13	25	65.8			15	23	60.5		
PS score											
0–1	41	20	21	51.2	1.7511	0.186	19	22	53.7	1.8421	0.175
2	46	16	30	65.2			28	18	39.1		

a Lymph node metastasis was present in all stage IIIB–IV patients, therefore, the values are not included in the table.

PI3K, phosphatidylinositol 3-kinase; p-Akt, phosphorylated-protein kinase B; SCC, squamous cell carcinoma; AdC, adenocarcinoma; PS, performance status; NSCLC, non-small cell lung carcinoma; TNM, tumor-node-metastasis.

**Table III:** Multivariate analysis of survival in stage IIIB–IV NSCLC.

Parameter^a^	Regression co-efficient	Standard error	Wald	HR (95% CI)	P-value
PI3K overexpression	0.762	0.291	6.860	2.143 (1.211–3.790)	0.009
p-Akt overexpression	0.689	0.347	3.948	1.991 (1.009–3.927)	0.047
Age, years (≥60 vs. <60)	−0.156	0.233	0.446	0.856 (0.542–1.351)	0.504
Gender (male vs. female)	−0.008	0.258	0.001	0.992 (0.598–1.646)	0.976
TNM stage (IIIb vs. IV)	1.566	0.313	24.982	4.788 (2.591–8.848)	0.001
Degree of differentiation (low-middle vs. high)	−0.013	0.241	0.003	0.987 (0.616–1.583)	0.963
PS score (0–1 vs. 2)	1.186	0.334	12.609	3.272 (1.701–6.296)	0.001

a Lymph node metastasis was present in all stage IIIB–IV patients, therefore, the values are not included in the table.

HR, hazards ratio; CI, confidence interval; PI3K, phosphatidylinositol 3-kinase; p-Akt, phosphorylated-protein kinase B; PS, performance status; NSCLC, non-small cell lung carcinoma; TNM, tumor-node-metastasis.

## References

[1] Molina JR, Yang P, Cassivi SD, Schild SE, Adjei AA (2008). Non-small cell lung cancer: epidemiology, risk factors, treatment, and survivorship. Mayo Clin Proc.

[2] Jemal A, Bray F, Center MM, Ferlay J, Ward E, Forman D (2011). Global cancer statistics. CA Cancer J Clin.

[3] Cantrell DA (2001). Phosphoinositide 3-kinase signaling pathways. J Cell Sci.

[4] Hennessy BT, Smith DL, Ram PT, Lu Y, Mills GB (2005). Exploiting the PI3K/AKT pathway for cancer drug discovery. Nat Rev Drug Discov.

[5] Sunayama J, Sato A, Matsuda K (2010). Dual blocking of mTor and PI3K elicits a prodifferentiation effect on glioblastoma stem-like cells. Neuro Oncol.

[6] Zhou J, Wulfkuhle J, Zhang H (2007). Activation of the PTEN/mTOR/STAT3 pathway in breast cancer stem-like cells is required for viability and maintenance. Proc Natl Acad Sci USA.

[7] Missiaglia E, Dalai I, Barbi S (2010). Pancreatic endocrine tumors: expression profiling evidences a role for AKT-mTOR pathway. J Clin Oncol.

[8] Al-Saad S, Donnem T, Al-Shibli K, Persson M, Bremnes RM, Busund LT (2009). Diverse prognostic roles of AKT isoforms, PTEN and PI3K in tumor epithelial cells and stromal compartment in non-small cell lung cancer. Anticancer Res.

[9] David O, Jett J, LeBeau H (2004). Phospho-Akt over expression in non-small cell lung cancer confers significant stage-independent survival disadvantage. Clin Cancer Res.

[10] Tang JM, He QY, Guo RX, Chang XJ (2006). Phosphorylated Akt over expression and loss of PTEN expression in non-small cell lung cancer confers poor prognosis. Lung Cancer.

[11] Tsao AS, McDonnell T, Lam S (2003). Increased phospho-AKT (Ser (473)) expression in bronchial dysplasia: implications for lung cancer prevention studies. Cancer Epidemiol Biomarkers Prev.

[12] Tsurutani J, Fukuoka J, Tsurutani H (2006). Evaluation of two phosphorylation sites improves the prognostic significance of Akt activation in non-small cell lung cancer tumors. J Clin Oncol.

[13] Shah A, Swain WA, Richardson D (2005). Phospho-Akt expression is associated with a favorable outcome in non-small cell lung cancer. Clin Cancer Res.

[14] Groome PA, Bolejack V, Crowley JJ (2007). IASLC International Staging Committee; Cancer Research and Biostatistics; Observers to the Committee; Participating Institutions: The IASLC Lung Cancer Staging Project: validation of the proposals for revision of the T, N, and M descriptors and consequent stage groupings in the forthcoming (seventh) edition of the TNM classification of malignant tumors. J Thorac Oncol.

[15] Shaw RJ, Cantley LC (2006). Ras, PI(3)K and mTOR signalling controls tumor cell growth. Nature.

[16] Pisick E, Jagadeesh S, Salgia R (2004). Receptor tyrosine kinases and inhibitors in lung cancer. ScientificWorldJournal.

[17] Brunet A, Bonni A, Zigmond MJ (1999). Akt promotes cell survival by phosphorylating and inhibiting a Forkhead transcription factor. Cell.

[18] Henshall DC, Araki T, Schindler CK (2002). Activation of bcl-2 associated death protein and counter-response of Akt within cell populations during seizure-induced neuronal death. J Neurosci.

[19] Duguay D, deBlois D (2007). Differential regulation of Akt, caspases and MAP kinases underlies smooth muscle cell apoptosis during aortic remodelling in SHR treated with amlodipine. Br J Pharmacol.

[20] Diehl JA, Cheng M, Roussel MF, Sherr CJ (1998). Glycogen synthase kinase-3b regulates cyclin D1 proteolysis and subcellular localization. Genes Dev.

[21] Sunters A, Madureira PA, Pomeranz KM, Aubert M, Brosens JJ, Cook SJ (2006). Paclitaxel-induced nuclear translocation of FOXO3a in breast cancer cells is mediated by c-Jun NH2-terminal kinase and Akt. Cancer Res.

[22] Wang L, Cao XX, Chen Q, Zhu TF, Zhu HG, Zheng L (2009). DIXDC1 targets p21 and cyclin D1 via PI3K pathway activation to promote colon cancer cell proliferation. Cancer Sci.

[23] Hahn-Windgassen A, Nogueira V, Chen CC, Skeen JE, Sonenberg N, Hay N (2005). Akt activates mTOR by regulating cellular ATP and AMPK activity. J Biol Chem.

[24] Zhang R, Xu Y, Ekman N (2003). Etk/Bmx transactivates vascular endothelial growth factor 2 and recruits phosphatidylinositol 3-kinase to mediate the tumor necrosis factor-induced angiogenic pathway. J Biol Chem.

[25] Balsara BR, Pei J, Mitsuuchi Y (2004). Frequent activation of Akt in non-small cell lung carcinomas and preneoplastic bronchial lesions. Carcinogenesis.

